# Computerized-Adaptive vs. Traditional Ratings of Depression and Suicidal Thoughts: An Assay Sensitivity Pilot Study in a Ketamine Clinical Trial

**DOI:** 10.3389/fpsyt.2021.602976

**Published:** 2021-04-07

**Authors:** Michael F. Grunebaum, J. John Mann, Hanga C. Galfalvy, Robert D. Gibbons

**Affiliations:** ^1^Department of Psychiatry, Columbia University Medical Center and New York State Psychiatric Institute, New York, NY, United States; ^2^Departments of Medicine and Public Health Sciences, University of Chicago, Chicago, IL, United States

**Keywords:** suicide assessment, depression scale, clinical trial, assay sensitivity, signal to noise

## Abstract

There is a public health need for improved suicide risk assessment tools. This pilot methodology study compared the assay sensitivity of computerized adaptive tests (CAT) of depression and suicidal ideation vs. traditional ratings in a randomized trial subgroup. The last 20 persons to enroll in a published ketamine trial in suicidal depression were studied. This subgroup received traditional and CAT ratings at baseline, 24 h post-infusion and follow-up week 2, 4, and 6: Hamilton Depression Rating Scale, Beck Depression Inventory, and Beck Scale for Suicidal Ideation vs. the CAT-Depression Inventory and CAT-Suicide Scale. Results showed larger effect sizes (ES) for CAT compared with traditional clinician-rated and self-report scales. Coefficients of variation for baseline measurements were lower for CAT compared with traditional scales. This is the first study to show that CAT may have greater assay sensitivity for treatment effects, particularly for suicidal ideation, compared with traditional clinician-rated and non-adaptive self-rated scales in a randomized trial. The findings suggest CAT can enable quick long-term follow-up assessments via cellphone, tablet, or computer while minimizing response bias due to repeated measurement of the same symptom items.

**Clinical Trial Registration:** www.ClinicalTrials.gov, identifier: NCT01700829.

## Introduction

The rising US suicide rate is a major public health problem (1) underscoring the importance of accurately assessing suicidal ideation. There are numerous rating scales for suicidal ideation and behavior, but little evidence to guide choice of a specific instrument (2). Simultaneously, there is a shift toward empirically derived and “interview independent tools” (3).

Advances in computerized adaptive testing (CAT) (4, 5) have improved the ability to precisely measure mental health constructs such as depression (4), anxiety (6), mania (7), and suicidality (8). CAT is generally based on an underlying statistical measurement model, item response theory (IRT), which is calibrated in advance in a large sample of patients with varying levels of the disorder of interest. From the complete data set comprised of responses to all of the symptom items in the “item bank,” CAT is simulated so that the correlation between a reduced set of adaptively administered items and the total item bank score is maximized (typically *r* > 0.90), and the number of adaptively administered items is minimized (typically an average of ~10 symptom items). As a result, the information from hundreds of items can be extracted using a much smaller set of adaptively administered items. This minimizes patient burden and staff effort, the latter limited to facilitating the participant's completing the CAT.

IRT-based CAT has been widely used in educational measurement, but its widespread use in mental health has been limited by the assumption of unidimensionality of the underlying constructs of interest. Generalizations of unidimensional IRT to multidimensional IRT (MIRT) (9–11) provide more appropriate model-based measurements for mental health constructs which are inherently multidimensional. CAT has been generalized to accommodate MIRT (4, 5). As an example, it was shown that the information contained in a 389-item bank of depressive symptoms could be adaptively measured using mean of 12 items in an average of 2 min, with a correlation of *r* = 0.95 with the item bank total score (4). By contrast, attempts at using traditional unidimensional IRT-based CAT for the adaptive measurement of depression have resulted in small (26 items) item banks (due to failure of the unidimensionality assumption), limiting the ability to measure the severity of depression across the entire underlying continuum (12).

Despite statistical demonstrations of speed and precision (7), convergent validity and greater test-retest reliability compared with established depression scales (4, 13), the validity of MIRT-based CAT for studying treatment efficacy has not been compared head-to-head with traditional instruments. We piloted such a comparison in the last 20 participants in a published, midazolam-controlled clinical trial (RCT) of intravenous ketamine for rapid reduction of suicidal thoughts in patients with major depressive disorder (MDD) (14). We compared the CAT-Suicide Scale (CAT-SS) (8) to the clinician-rated Beck Scale for Suicidal Ideation (SSI) (15). We compared the CAT-Depression Inventory (CAT-DI) (4) to the 17-item Hamilton Depression Rating Scale (HAM-D) (16) and the self-reported Beck Depression Inventory (BDI) (17) for the 24 h post-infusion primary endpoint (day 1) of the blinded portion of the study. We also compare the rates of change in these measures (excluding the BDI) over the 6 week open treatment observational phase of the study.

## Methods

### Participants

Main clinical trial results were published (14). Briefly, eligible participants in the parent RCT were 18–65 years old, with a diagnosis of MDD, in a current major depressive episode (MDE), diagnosed using the Structured Clinical Interview for DSM-IV Axis I Disorders (18), with a score >15 on the 17-item HAM-D and a score ≥4 on the SSI. Participants were recruited as outpatients, inpatients, or from the emergency department, and were admitted to a research unit at the New York State Psychiatric Institute for the infusion phase of the study. Main exclusion criteria included unstable medical or neurological illness, significant electrocardiographic abnormality, pregnancy or lactation, current psychosis, history of ketamine abuse or dependence, other drug or alcohol dependence within the past 6 months, suicidal ideation due to binge substance use or withdrawal, lack of capacity to consent, and inadequate understanding of English. Patients continued their current psychiatric medications at stable doses during the infusion phase except for benzodiazepines which were discontinued at least 24 h before the infusion. The study protocol was approved by the Institutional Review Board of the New York State Psychiatric Institute, and written informed consent was obtained from all participants. The opportunity to compare assay sensitivity of the CAT-SS and CAT-DI head-to-head with the HAM-D, BDI and SSI occurred only in time for the final 20 (of 80) participants in the clinical trial, so this subgroup is the sample for this analysis.

### Intervention

Participants were randomly assigned to intravenous racemic ketamine hydrochloride at 0.5 mg/kg (*N* = 9) or midazolam at 0.02 mg/kg (*N* = 11), in 100 mL normal saline infused over 40 min with frequent vital signs monitoring. At day 1, non-remitters (remission defined as SSI at least 50% below baseline and less than the study eligibility threshold of 4) were un-blinded and those allocated to midazolam received an open label ketamine infusion.

### Outcome Measures

CAT measures, using proprietary software (CAT-MH^TM^, Adaptive Testing Technologies, Chicago, IL, www.adaptivetestingtechnologies.com), target a range of psychiatric disorders and are optimized for various populations. They became available to pilot only for the last 20 participants in our ketamine trial, and were assessed at baseline, day 1, and follow-up weeks 2, 4, and 6 as add-ons to trial ratings. This permitted a head-to-head assay sensitivity comparison between the traditional scales (SSI, HAM-D, BDI) and the CAT-SS and CAT-DI ratings. In general, a CAT-MH™ test is a type of self-report that tailors item administration to a given individual in real time by learning that person's severity from early item responses using a pre-calibrated item bank. After a few items, the CAT-MH™ quickly targets the remaining items in the test to that person's severity level on that occasion.

The CAT-SS reproduces the information in a 111 item bank that provides a crosswalk between symptoms of depression, anxiety and suicidality, using an average of 10 items in <2 min, while maintain a correlation of *r* = 0.96 with the total 111 item bank score. The CAT-SS was validated against the clinician-administered Columbia Suicide Severity Rating Scale (C-SSRS) and demonstrated sensitivity (Se) of 1.0 and specificity (Sp) of 0.95 for ideation or worse (kappa 0.81); active ideation Se = 1.0, Sp = 0.95; suicide alert Se = 1.0, Sp = 0.89; and lifetime attempt Se = 0.58 and Sp = 0.88 for the CAT-SS high risk vs. low risk groups (8).

The CAT-DI reproduces the information in a bank of 389 items using adaptive administration of 12 items in ~2 min while maintaining a correlation of 0.95 with the total bank score. In terms of convergent validity, correlations were *r* = 0.81 with the PHQ-9, *r* = 0.75 with the HAM-D, and *r* = 0.84 with the CES-D. In general, the distribution of scores between the diagnostic categories showed greater overlap (i.e., less diagnostic specificity), greater variability, and greater skewness for these other scales relative to the CAT-DI. A thresholded CAT-DI yielded Se of 0.92 and Sp of 0.88 for a diagnosis of major depressive disorder (4).

The *a priori* primary outcome of the trial was at day 1, during the blinded portion of the study. Longitudinal ratings during the 6-week observational follow-up phase, during which all patients received optimized clinical pharmacotherapy without a control group, was a secondary outcome. To compare the CAT-DI to a traditional self-report measure, we used data from the BDI which was only assessed at baseline and day 1.

### Statistical Methods

Two analyses of these data were conducted. First, we compared the changes from baseline to day 1 post-infusion between participants randomized to ketamine vs. midazolam using a linear mixed-effects regression model (19) with main effects of drug (ketamine = 1), day (24 h post-infusion = 1) and the drug by day interaction. The data were clustered within subjects and the random effects included intercept and day. The drug by day interaction was the key effect of interest. Second, we pooled all data for subjects treated with ketamine during the blinded and open-label phases of the study. The latter included non-remitters to the randomized infusion who had received midazolam and subsequently had an open ketamine infusion (14). These data were analyzed using a linear mixed-effects regression model with a main effect of day (coded 0, 1, 14, 28, 42 days). The main effect in that model would describe the linear rate of change over the 6-week study. Inspection of the data revealed that the time trends were not linear in day, but were approximately linear for the square root of day (0.00, 1.00, 3.74, 5.29, 6.48), and thus the model was modified to include the square root of days instead. Each analysis was performed separately for each of the suicidal ideation and depression severity endpoints. In addition, the drug by day interaction at 24 h and the estimated change at 42 days were described as standardized effect sizes and the sample size required to achieve power of 80% for a Type I error rate of 5%, was computed since this pilot study was based on a small subgroup. Baseline coefficients of variation (SD/Mean) were computed for each of the measures. Given the small subgroup sample, our focus is on the magnitudes of the effects and to a lesser extent the statistical significance of those results.

## Results

### Pilot Subgroup

Table 1 summarizes baseline demographic and clinical characteristics of the subgroup (*N* = 20) who received pilot CAT ratings in addition to standard scales as compared to those (*N* = 60) who received only the latter. At baseline, the groups did not differ in age, sex, race, education, employment, marital status, prior psychiatric hospitalization, alcohol or substance use disorder history, prior suicide attempt, severity of depression (HAM-D) or suicidal ideation (SSI), age at onset of first MDE, or body mass index. The only differences were that the CAT subgroup had a shorter median length of current MDE but more lifetime MDEs. Overall, at baseline, the CAT subgroup was comparable to the rest of the study sample, and in particular, on the clinician-rated scales that are the focus of this pilot study.

**Table 1:** Baseline characteristics of patients with major depressive disorder and clinically significant suicidal ideation given a single infusion of ketamine or midazolam according to Computerized Adaptive Test (CAT) status^a^.

	**CAT Sub-sample (*****N*** **=** **20)**		**Standard ratings (*****N*** **=** **60)**				
**Variable^**b**^**	***N***	**%**	***N***	**%**	**χ^**2**^**	**df**	***p***
Female sex	10	50	38	63	1.11	1	0.292
White race	19	95	54	90			0.673^c^
Married	3	15	11	18			1.000^c^
Currently employed	8	40	17	28	0.95	1	0.330
Prior psychiatric hospitalization	14	70	42	70	0.00	1	1.000
Prior suicide attempt	12	60	27	45	1.35	1	0.245
Alcohol or substance use disorder history	15	75	45	75	0.00	1	1.000
**Variable**	***N***	**Mean** **±** **SD**	***N***	**Mean** **±** **SD**	***t***	**df**	***p***
Age	20	36.6 ± 11.6	60	40.5 ± 13.5	1.18	78	0.242
Total years of education	20	16.1 ± 2.4	59	15.8 ± 2.7	−0.32	77	0.747
Scale for suicidal ideation^d^	20	15.0 ± 6.6	60	14.9 ± 6.7	−0.02	78	0.985
Hamilton depression rating scale (17 item)^e^	20	22.2 ± 5.1	60	22.5 ± 4.0	0.27	78	0.789
Beck depression inventory^f^	20	33.5 ± 7.1	54	32.6 ± 8.5	−0.38	72	0.704
**Variable**		**Median (IQR)**		**Median (IQR)**	**U**^**g**^		***p***
Length of current episode (weeks truncated at 104)	20	42.5 (12–82.5)	53	72 (24–104)	371.0		0.043
Age of onset of first major depressive episode (years)	19	16 (12–20)	57	16 (11–25.5)	520.5		0.801
Lifetime number of major depressive episodes including current (truncated at 30)	18	17.5 (2.8–30)	53	4 (1–8)	640.0		0.028
Body mass index	20	28.3 (23.2–32.2)	60	25.4 (22.7–30.6)	673.5		0.414

a *All subjects received standard rating scales; the last 20 subjects to enroll additionally received pilot CAT ratings of depressive symptoms and suicidal ideation*.

b *Assessed with our research Baseline Clinical-Demographic form unless otherwise noted*.

c *Fisher's Exact Test*.

d *Score ranges from 0 to 38, with higher scores indicating greater symptom severity*.

e *Score ranges from 0 to 53, with higher scores indicating greater symptom severity*.

f *Score ranges from 0 to 63, with higher scores indicating greater symptom severity*.

g *Mann-Whitney U*.

### Variability

For the two suicidal ideation measures, the coefficients of variation (CV) at baseline were 44% for the SSI and 18% for the CAT-SS. For the depression measures, the CVs were 23% for the HAM-D, 22% for the BDI, and 19% for the CAT-DI.

### Twenty Four Hour Blinded Phase Treatment Response

#### Suicidal Ideation

The estimated average baseline SSI score in midazolam treated patients in this subgroup was 17.44 compared with 11.55 in the ketamine group. The midazolam treated patients decreased by 7.00 points on day 1 post-infusion (to a score of 10.44) and the ketamine treated subjects decreased by 5.67 points (to a score of 5.88). For the SSI, the drug by day interaction (in the original score metric) was 1.33 points on the scale (*p* = 0.62) indicating slightly less change with ketamine. Since in this subgroup there was no benefit of ketamine over midazolam, the effect size is zero. While the post-treatment score for ketamine was lower than midazolam, this was accounted for by the larger baseline imbalance.

In contrast, for the CAT-SS, the drug by day interaction was −5.98 (*p* = 0.44) indicating a 54% greater decrease in suicidal ideation for ketamine relative to midazolam. The estimated average CAT-SS score at baseline among midazolam treated patients was 68.10 compared with ketamine treated subjects having a baseline score of 65.18. The midazolam treated patients decreased by 10.98 points on day 1 post-infusion (to a score of 57.12) and the ketamine treated subjects decreased by 16.96 points (to a score of 48.22). This represents an effect size of 0.40.

#### Depression Severity

For the HAM-D, the drug by day interaction was −3.78 (*p* = 0.19) indicating a 92% greater decrease in depressive severity for ketamine relative to midazolam. The estimated average HAM-D score at baseline among midazolam treated patients was 21.33 compared with ketamine treated subjects having a baseline score of 22.00. The midazolam treated patients decreased by 4.11 points on post-treatment day 1 (to a score of 17.22) and the ketamine treated subjects decreased by 7.89 points (to a score of 14.11). This represents an effect size of 0.58.

For the BDI, the drug by day interaction was −0.89 (*p* = 0.83) indicating an 8% greater decrease in depressive severity for ketamine relative to midazolam. The estimated average BDI score at baseline among midazolam treated patients was 34.56 compared with ketamine treated subjects having a baseline score of 32.56. The midazolam treated patients decreased by 11.44 points on post-treatment day 1 (to a score of 23.12) and the ketamine treated subjects decreased by 12.33 points (to a score of 20.23). This represents an effect size of 0.09.

For the CAT-DI, the drug by day interaction was −11.72 (*p* = 0.14) indicating a 106% greater decrease in depressive severity for ketamine relative to midazolam. The estimated average CAT-DI score at baseline among midazolam treated patients was 77.92 with ketamine treated subjects having a baseline score of 77.15. The midazolam treated patients decreased by 10.99 points on day 1 post-infusion (to a score of 66.93) and the ketamine treated subjects decreased by 22.71 points (to a score of 54.44). This represents an effect size of 0.71.

### Six-Week Observational Follow-up

#### Suicidal Ideation

**Figure 1:**
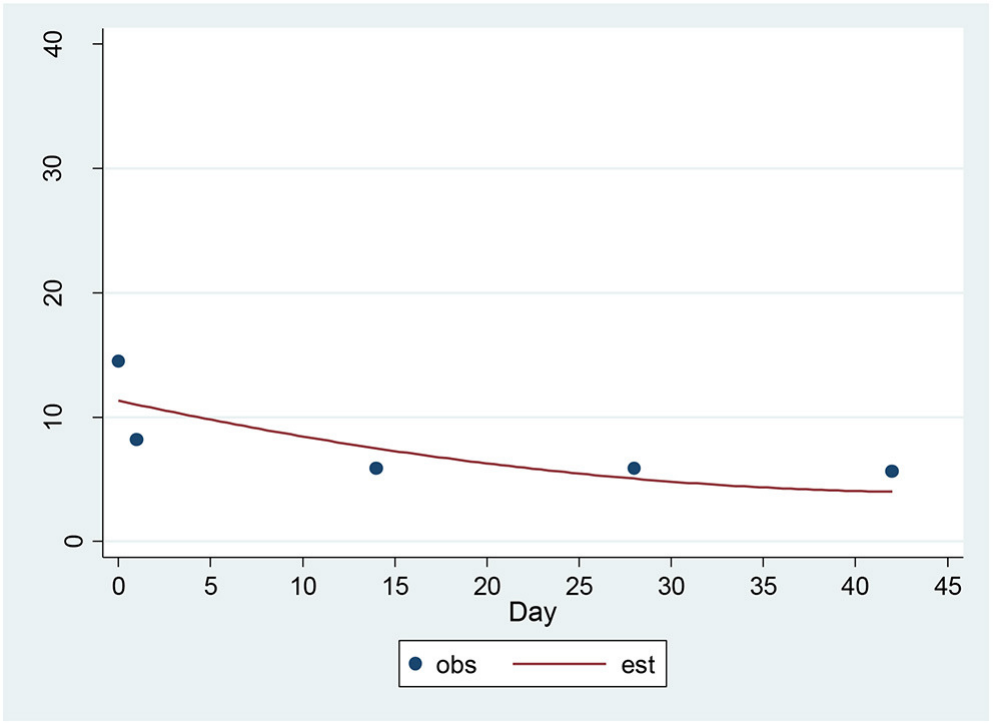
Observed and Estimated Time Trend – Beck SSI^a^. ^a^SSI, Clinician rated Scale for Suicidal Ideation.

For the SSI, a significant decrease of 1.22 units per sqrt(day) (*p* = 0.0004) was found (Figure 1). The estimated baseline score was 11.79 and the estimated day 42 score was 3.88. This represents an effect size of 0.93 SD units, which should be detected with power of 80% (confidence = 95%) in a sample of size 31.

**Figure 2:**
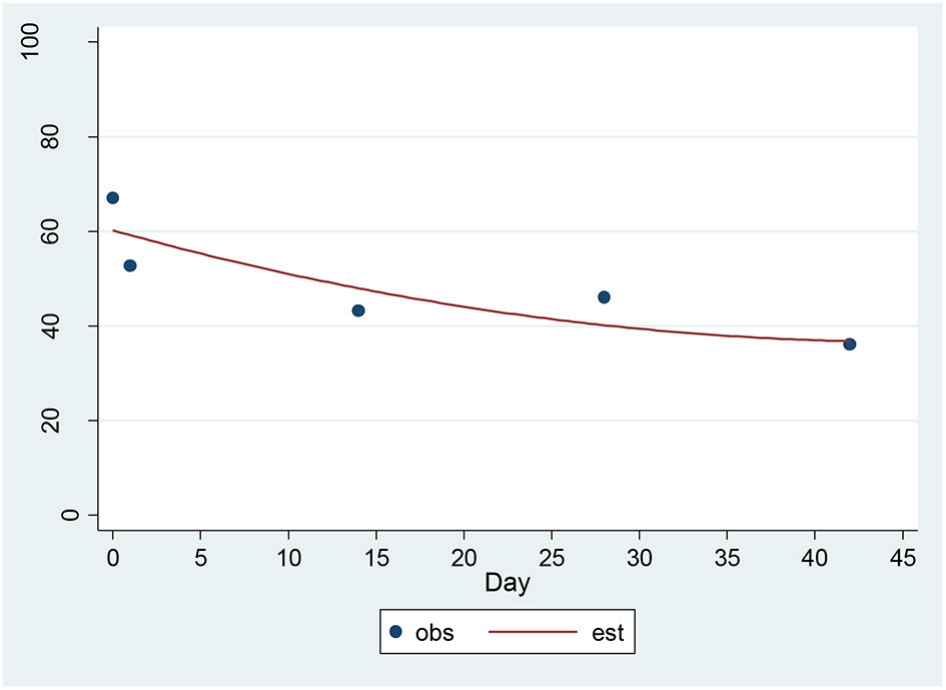
Observed and Estimated Time Trend – CAT-SS^a^. ^a^CAT-SS, Computerized Adaptive Test-Suicide Scale.

For the CAT-SS, a significant decrease of 3.89 units per sqrt(day) (*p* = 0.00001) was found (Figure 2). The estimated baseline score was 61.74 and the estimated day 42 score was 36.47. This represents an effect size of 1.14 SD units, which should be detected with power of 80% (confidence = 95%) in a sample of size 21. The SSI and the CAT-SS were correlated with *r* = 0.60 (*p* = 0.003) at baseline.

#### Depression Severity

**Figure 3:**
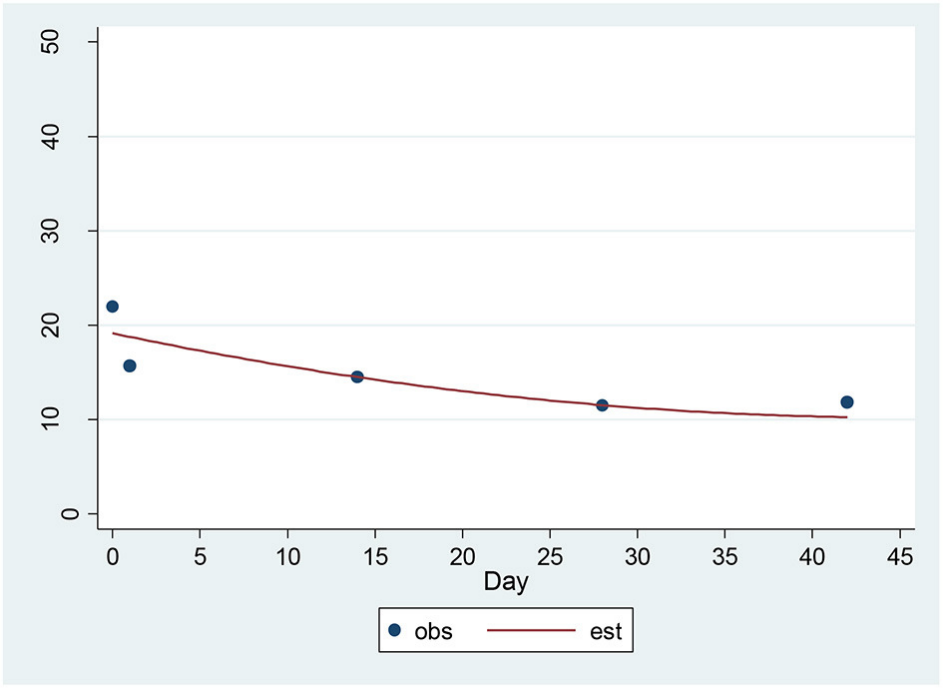
Observed and Estimated Time Trend – 17-item HAM-D^a^. ^a^HAM-D, 17-item Hamilton Depression Rating Scale.

For the HAM-D, a significant decrease of 1.48 units per sqrt(day) (*p* = 0.00001) was found (Figure 3). The estimated baseline score was 19.73 and the estimated day 42 score was 10.20. This represents an effect size of 1.39 SD units, which should be detected with power of 80% (confidence = 95%) in a sample of size 16.

**Figure 4:**
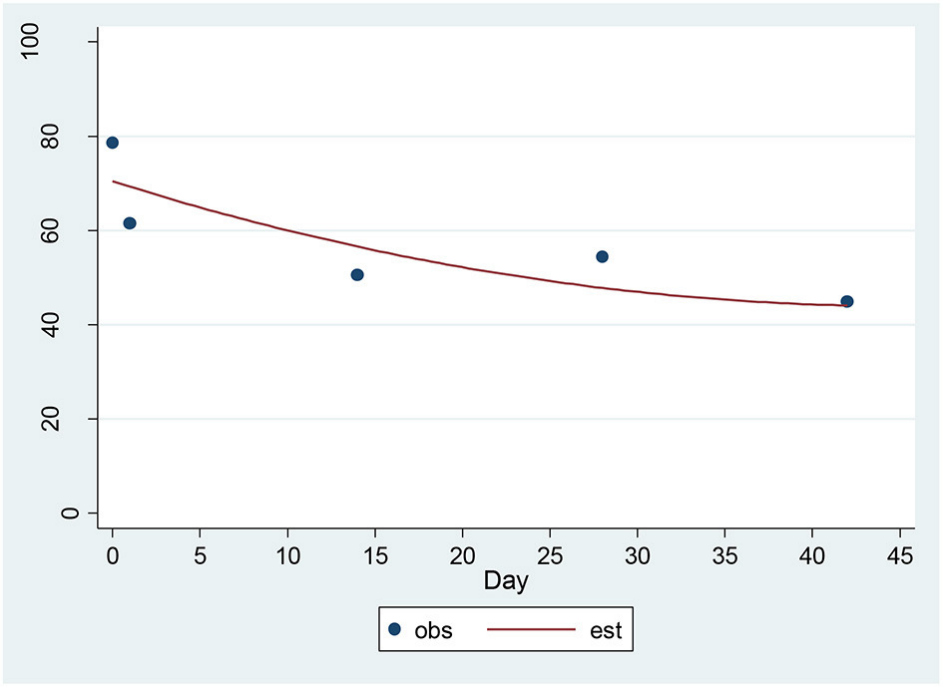
Observed and Estimated Time Trend – CAT-DI^a^. ^a^CAT-DI, Computerized Adaptive Test-Depression Inventory.

For the CAT-DI, a significant decrease of 4.36 units per sqrt(day) (*p* = 0.00001) was found (Figure 4). The estimated baseline score was 71.95 and the estimated day 42 score was 43.70. This represents an effect size of 1.24 SD units, which should be detected with power of 80% (confidence = 95%) in a sample of size 17. The 17-item HAM-D and the CAT-DI were correlated with *r* = 0.72 (*p* = 0.0002) at baseline.

## Discussion

Results of this pilot study suggest that computerized adaptive self-reports for suicidal ideation and depression have equal or greater assay sensitivity compared to traditional self- or clinician-rated measures. For the clinician-rated SSI, in this 20 patient subgroup, the same ~5 point difference at the end of the study (10.44 midazolam vs. 5.88 ketamine) was observed as in the original study with all 80 patients (14); however, in this subsample, there was an imbalance at baseline between ketamine and midazolam of −5.89 units which outweighed this. The analogous baseline difference in the full study sample was 1.4 points, which was not statistically significant (14). In contrast, this baseline imbalance was not observed for the CAT-SS, where a 54% reduction in suicidal ideation with ketamine treatment was found, yielding an effect size of 0.40.

Neither the HAM-D or the CAT-DI showed any appreciable baseline imbalance in this subsample; however, the CAT-DI detected a 106% estimated decrease in severity with ketamine treatment and the HAM-D a 92% decrease with effect sizes of 0.71 and 0.58 SD units, respectively. The self-report BDI detected an 8% estimated decrease in severity with ketamine treatment vs. the 106% decrease observed with the CAT-DI, with effect sizes of 0.09 vs. 0.71 SD units, respectively.

In the combined longitudinal data which included an open-label ketamine treatment for patients who did not have remission of suicidal ideation to midazolam, significant reductions in both suicidal ideation and depression severity for both traditional and computerized-adaptive measures were observed. However, for suicidal ideation, the effect sizes were larger for the CAT-SS (ES = 1.136) vs. the SSI (ES = 0.93) which can be detected with power of 80% in samples of size 21 vs. 31, respectively. By contrast, for depression severity, performance of the HAM-D and CAT-DI was similar with effect sizes of 1.39 vs. 1.24, respectively, requiring sample sizes for 80% power of 16 vs. 17, respectively.

The greater differences observed for the CAT-SS and SSI are reflected in their lower correlation of *r* = 0.60 vs. the HAM-D and the CAT-DI which were correlated *r* = 0.72 at baseline. The performance of the CAT-SS in this small, exploratory study suggests a more favorable signal-to-noise ratio compared with the clinician-rated SSI, a result deserving replication in a larger sample.

It is possible that the inclusion of un-blinded data during the longitudinal follow-up had a greater effect on clinician ratings than on computerized self-report ratings and this may have accounted for larger differences in effect sizes observed at day 1 during the blinded phase of the study. However, the difference in effect size at day 1 was larger between the CAT-DI vs. the self-report BDI than the HAM-D. The BDI was not assessed during the follow-up phase. Of course, these 24-h effect sizes are for differential treatment related effects, and in the longitudinal follow-up phase they are restricted to rates of change in patients, all but two of whom received a ketamine infusion. Of the 11 patients randomized to midazolam, only two were responders, and 9 non-responders at day 1 received an open ketamine infusion usually the following day. It is also possible that the distribution of the SSI which has a large mass of zeroes for non-suicidal patients made it more difficult to detect change than for the CAT-SS which has a more continuous distribution because it can measure suicidal propensity in individuals who are not yet suicidal. This is a further advantage of the CAT-SS over traditional suicidality scales.

A limitation of this study is the fact that CAT measures became available in time to pilot test only in the final 20 participants in the 4-fold larger parent trial. Nonetheless, the CAT subgroup was comparable to the other 60 subjects across a range of baseline characteristics, including the key study measures. The CAT subgroup had shorter current MDE but more past MDEs, but both differences would be non-significant with Bonferroni correction. Another limitation is that the open ketamine infusion received by 9/11 midazolam-randomized non-responders creates, in effect, a new baseline. Nevertheless, the effect sizes for both suicidal ideation and depression severity were appreciably larger for the computerized adaptive measurements during the blinded phase of the study and statistically significant compared to the traditional clinician-rated SSI, HAM-D, and self-report BDI.

Advantages of CAT include the potential for cloud-based administration wherever a study participant has internet access. In addition, median administration time for completion of both CATs in this pilot study was 2:29 min (interquartile range 1:55, 3:30 min), an order of magnitude less than for the comparable clinician ratings. This could enable long-term follow-up assessments where patients can complete a brief CAT scale via cellphone, tablet or computer. Second, since the same items are not repeated over longitudinal assessments, there is no response bias due to repeated measurement of the same symptom items. Increased testing frequency will lead to increased statistical power to detect clinically important treatment related effects.

Results of this pilot study show that computerized adaptive self-reports for suicidal ideation and depression had overall greater assay sensitivity compared to traditional clinician-rated and self-report scales in a small subgroup from a published ketamine trial. Replication in larger studies would represent an advance - especially in the measurement of suicidal ideation where innovation is particularly needed (3) - for both research and clinical care.

## Data Availability Statement

The raw data supporting the conclusions of this article will be made available by the authors, without undue reservation.

## Ethics Statement

The studies involving human participants were reviewed and approved by New York State Psychiatric Institute Institutional Review Board. The patients/participants provided their written informed consent to participate in this study.

## Author Contributions

MG: was awarded the NIMH grant and was principal investigator of the parent clinical trial study which collected these data, participated in analyzing and interpreting the data, drafting and editing the manuscript. JM: a co-investigator on the grant and parent clinical trial, participated in incorporating CAT ratings into the trial, interpreting the data, drafting and editing the manuscript. HG: statistician on the parent clinical trial, participated in CAT data analysis and interpretation, drafting and editing the manuscript. RG: lead developer of CAT ratings, collaborated on incorporating CAT ratings into the parent clinical trial for pilot testing, principal analyst of CAT data for this study, participated in drafting and editing the manuscript. All authors contributed to the article and approved the submitted version.

## Conflict of Interest

RG was a founder of Adaptive Testing Technologies, which licenses use of the CAT-MH™. The terms of this arrangement have been reviewed and approved by the University of Chicago in accordance with its conflict of interest policies. JM received royalties from the Research Foundation for Mental Hygiene for commercial use of the Columbia Suicide Severity Rating Scale which was not used in this study. The remaining authors declare that the research was conducted in the absence of any commercial or financial relationships that could be construed as a potential conflict of interest.
